# Revealing High Oxygen Evolution Catalytic Activity of Fluorine-Doped Carbon in Alkaline Media

**DOI:** 10.3390/ma12020211

**Published:** 2019-01-10

**Authors:** Jeheon Kim, Tomohiro Fukushima, Ruifeng Zhou, Kei Murakoshi

**Affiliations:** 1 Department of Chemistry, Faculty of Science, Hokkaido University, Sapporo 060-0810, Japan; jeheonkim@frontier.hokudai.ac.jp (J.K.); tfuku@sci.hokudai.ac.jp (T.F.); 2 Institute for International Collaboration, Hokkaido University, Sapporo 060-0815, Japan; ruifeng.zhou@oia.hokudai.ac.jp

**Keywords:** oxygen evolution reaction, carbon materials, kinetic product analysis

## Abstract

Oxygen evolution reactions (OER) are important reactions for energy conversion. Metal-free carbon-based catalysts potentially contribute to the catalytic materials for OER. However, it has been difficult to understand the intrinsic catalytic activity of carbon materials, due to catalyst decomposition over the course of long-term reactions. Here, we report high oxygen evolution reaction catalytic activity of F-doped carbon in alkaline media. Intrinsic OER activity was evaluated from a combination of measurements using a rotating disk electrode and O_2_ sensor. The F-doped carbon catalyst is a highly active catalyst, comparable to state-of-the-art precious-metal-based catalysts such as RuO_2_.

## 1. Introduction

The oxygen evolution reaction (OER) in water splitting reactions is an important reaction for energy conversion and storage [1]. High overpotential is generally required to promote the reaction kinetics of the 4-electron reaction for the oxidation of hydroxide to oxygen in alkaline conditions [2]. Researchers have studied OER activity for metal oxide thin films or nanoparticles on conductive substrates. Ruthenium oxide (RuO_2_) is a state-of-the-art catalyst with high stability [3]. However, precious-metal-based catalysts are limited by their cost and abundance. Recently, carbon materials have been highlighted. We recently reported that semi-ionic C–F bonds in fluorine-doped carbon (F–carbon) enhance the catalysis for OER in alkaline media [4]. Comparable catalysts have been reported for carbon based catalysis of OER [5,6,7,8]. However, in general, due to the highly oxidative conditions of OER, catalyst degradation is problematic for analysis of intrinsic OER activity [9,10]. In order to investigate OER kinetics, rates of reaction for OER ($r_{\mathrm{OER}}$) should be evaluated by appropriate analytical methods. 

The most commonly used method is electrochemical experimentation, which is actually useful if the current density is only derived from a single faradaic electrocatalytic OER process. Rotating ring disk electrode (RRDE) systems can be utilized for evaluation of the kinetics of OER [11,12]. Evolved O_2_ is measured on a ring electrode by monitoring the current of the oxygen reduction reaction at a constant potential, which is proportional to the amount of O_2_ evolved from the disk electrodes. However, we assume that the ring current is sensitive to catalyst corrosion or formation of O_2_ bubbles from the disk electrode, leading to difficulty evaluating OER from the disk electrode. The other evaluation method is differential electrochemical mass spectrometry (DEMS) during cyclic voltammetry [13]. DEMS records the ion current of species on porous electrodes. However, DEMS requires sufficient catalyst for detection at about 10 ppb [14]. A fluorescence-based O_2_ sensor, which detects fluorescent quenching by O_2_, has been used for evaluation of OER rate in a gas-tight electrochemical cell [15]. An O_2_ sensor was placed in the headspace over the electrolyte. The O_2_ sensor method takes a long time, due to gas exchange between the gas and solution phases. Actually, the fluorescent O_2_ detector is quite sensitive even for concentration levels of O_2_ as low as a few ppb with ignorable noise level. Therefore, O_2_ detection methods in solution could be a candidate for the evaluation of OER catalytic activity even for carbon materials.

Here, we report high catalytic activity for our F–carbon, as evaluated based on electrode kinetics. This analytical method is based on the combination of measurement of faradic current using a rotating disk electrode (RDE), and detection of evolved O_2_ by fluorescent sensor, which is placed in the electrolyte and close to the catalyst. Because of shear flow induced by rotation of the RDE, in situ and real-time measurement is possible by O_2_ sensor. We constructed a kinetic model of the OER reaction by analysis of in situ monitoring of O_2_ concentration. This methodology revealed that our F–carbon catalyst is comparable to the precious metal oxide.

## 2. Materials and Methods

### 2.1. Sample Preparation

Nafion (5% dispersion solution), Ketjen black (EC-600JD), and RuO_2_ (~99%) were purchased from Wako Pure Chemical Industries, LTD. (Osaka, Japan), Lion Special Chemicals Co., LTD. (Tokyo, Japan), and Sigma-Aldrich (St. Louis, MO, USA), respectively. Nafion (400 μL) and Ketjen black (10 mg) were dispersed in methanol (10 mL) using an ultrasonication homogenizer (UH-50F, SMT, Tokyo, Japan) at 50 W and 20 kHz for 30 min. After drying overnight at 60 °C, the mixture was pyrolyzed in Ar (200 sccm) for 1 h at 600 °C. F–carbon has 2 types of C–F bond: covalent (689.0 eV) and semi-ionic (687.0 eV), as shown in X-ray photoelectron spectroscopy (XPS, JEOL, Tokyo, Japan) in Figure S1. The atomic ratios of covalent and semi-ionic C–F bonds are 1.6% and 3.9%, respectively. The RDE was prepared as follows. The sample (1 mg) was dispersed in ethanol (40 μL) and deionized water (160 μL) using an ultrasonic homogenizer for 10 min. Nafion (2 μL) was then added to the composite ink and dispersed by ultrasonic homogenizer for 1 min. The catalyst (8.3 μL) was loaded onto a glassy carbon disk electrode (5 mm diameter). Catalyst loading was calculated as 0.2 mg cm^−2^.

### 2.2. Electrochemical Characterization

The electrochemical cell was equipped with an O_2_ sensor (Pyroscience OXROB10, BAS, Tokyo, Japan), RDE working electrode, Pt counter electrode, and a reversible hydrogen electrode (RHE) as the reference electrode. The distance between the dissolved O_2_ detector and working electrode was fixed at 5 mm. The electrochemical cell is schematically shown in Figure S2. To minimize the dissolved O_2_ before measurements, the electrolyte was bubbled with Ar gas until the dissolved O_2_ concentration was stable for 10 min. The background concentration was subtracted and defined as 0 ppb. To evaluate the effect of pH on OER on the F–carbon, the pH of the KOH alkaline solution was set at 12, 13, 13.5, and 14 by pH meter (Horiba, 9625-10D, Kyoto, Japan). Chronoamperometry (current density versus time) of F–carbon was stepped at 1.5 V, 1.53 V, 1.56 V, 1.59, and 1.62 V for 1 min. In case of the RuO_2_, chronoamperometry was stepped at 1.4 V, 1.43 V, 1.46 V, 1.49 V, and 1.52 V for 1 min. Rotation speed of the RDE was maintained at 1600 rpm. Potential versus log O_2_ concentration curves were estimated by increment of O_2_ concentration per 1 min for potential. Tafel plot (potential versus log current density) was plotted by averaged current density for 1 min at given potentials.

### 2.3. Theoretical Calculation

Density functional theory (DFT) calculations were carried out using Gaussian 03 software, at the B3LYP level of DFT with 6-31G** basis sets. The molecular model for nanographene was treated as C_42_H_16_. The model structure was constructed using the Avogadro package, and structure was optimized by thermal quenching method. Thereafter, initial structure of molecular model was optimized using “opt” command in Gaussian. Atomic coordinates are shown in appendix (Supplementary Materials).

## 3. Results and Discussion

### 3.1. Evaluation of pH-Dependent Tafel Slopes from Electrochemical Measurement and O_2_ Sensor

**Figure 1:**
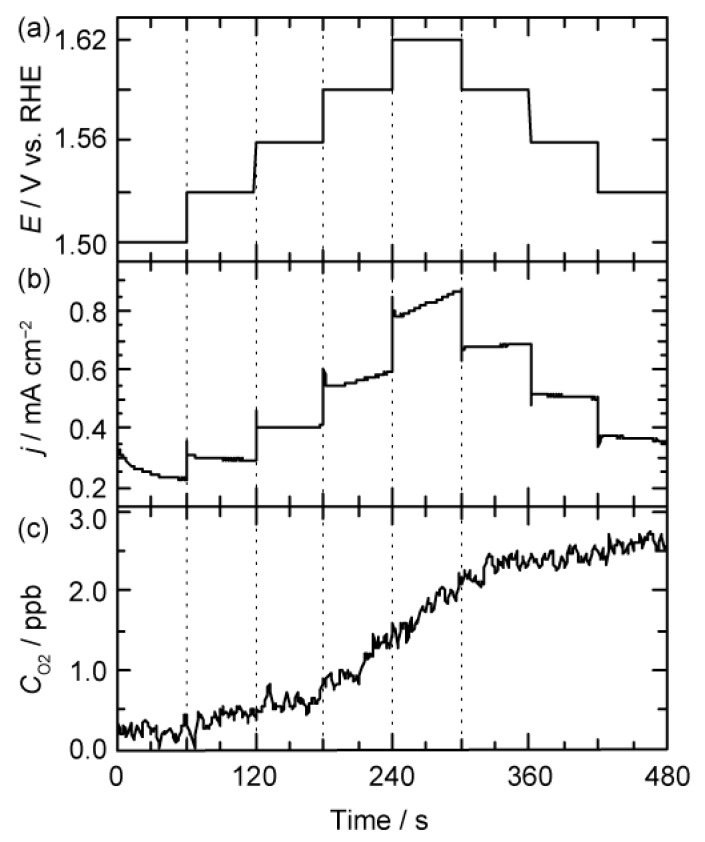
OER from F–carbon in 0.1 M KOH solution (pH = 13). (**a**) Applied potential (*E*), (**b**) current density ($j_{\mathrm{total}}$), and (**c**) O_2_ concentration ($C_{O_{2}}$ ) were plotted versus time.

We first confirmed the relationship between evolved O_2_ relative concentration ($C_{O_{2}}$) and total current density ($j_{\mathrm{total}}$), using RuO_2_ as model catalyst. Disk electrodes were rotated at 1600 rpm, and potentiostatic polarization was conducted in 0.1 M KOH aqueous solution (pH = 13) to induce OER. As a result, positive polarization of electrochemical potential led to increases in both $j_{\mathrm{total}}$ and O_2_ concentration $C_{O_{2}}$ (see Figure S3). Importantly, the slope of O_2_ concentration for time, i.e., rate for OER ($r_{\mathrm{OER}}$ = d$C_{O_{2}}$/d*t*), was increased with increment of $j_{\mathrm{total}}$. This result suggests that there is a positive correlation between $r_{\mathrm{OER}}$ and $j_{\mathrm{total}}$ using RDE with the O_2_ sensor method. With the aid of successive demonstrations of our experimental setup, we prepared and characterized the F–carbon electrode according to our previous work. The sample was characterized by XPS (see Figure S1) [4,16]. Figure 1 shows the plot of time versus applied potential, $j_{\mathrm{total}}$, and $C_{O_{2}}$, recorded in 0.1 M KOH aqueous solution (pH = 13). As discussed in the RuO_2_ experiment, $C_{O_{2}}$ increased as the electrochemical potential, *E*, increased. On the other hand, forward and backward traces for $j_{\mathrm{total}}$ and $r_{\mathrm{OER}}$ were distinct. For example, $v_{O_{2}}$ at 1.59 V in the forward trace (0.011 ppb s^−1^, 180–240 s) was twice as high as in the backward trace (0.0047 ppb s^−1^, 360–420 s). In addition to that, $j_{\mathrm{total}}$ was not stable during the potentiostatic polarization. On the other hand, $r_{\mathrm{OER}}$ showed linearity under potentiostatic polarization. This observation of $j_{\mathrm{total}}$ and $r_{\mathrm{OER}}$ suggests that $j_{\mathrm{total}}$ is derived from OER, as well as catalyst oxidation after the initiation of OER.

**Figure 2:**
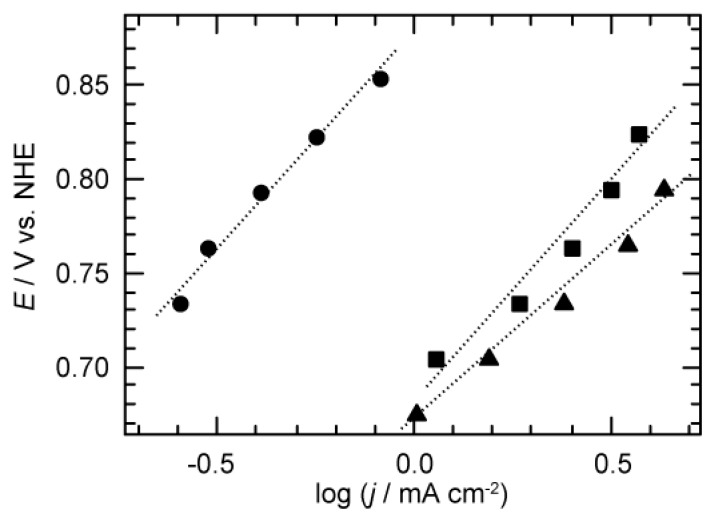
Tafel plot of logarithm total current density (log($j_{\mathrm{total}}$)) versus electrochemical potential (*E*) under oxygen evolution condition from F–carbon. The data were recorded in pH 13 (circle), pH 13.5 (square) and pH 14 (triangle). Current density was averaged throughout at each potential step.

We investigated effect of pH on the observed current density. Figure 2 shows the Tafel plot of OER from F–carbon by plotting log($j_{\mathrm{total}}$) and *E* versus normal hydrogen electrode (NHE) scale. We averaged $j_{\mathrm{total}}$ in each duration for potentiostatic polarization, because of the instability of the observed current density during chronoamperometry (see Figure S4). Importantly, $j_{\mathrm{total}}$ showed different behavior depending on pH of the KOH aqueous solution (pH = 12–14). In the case of pH 12, the current density did not show apparent increments, regardless of the applied potential. Increases in pH of more than 13 led to the increase of $j_{\mathrm{total}}$. In the case of pH 13.5 and 14, the values of $j_{\mathrm{total}}$ were almost of a similar order. The Tafel slopes, ∂*E*/∂log($j_{\mathrm{total}}$), varied depending on pH of solution, as shown in Figure 2. Tafel slopes at pH 13, pH 13.5, and pH 14 were 227 mV dec^−1^, 225 mV dec^−1^, and 183 mV dec^−1^, respectively. The relatively high Tafel slopes observed imply that the current density includes contributions from additional electrochemical side reactions such as catalyst degradation.

**Figure 3:**
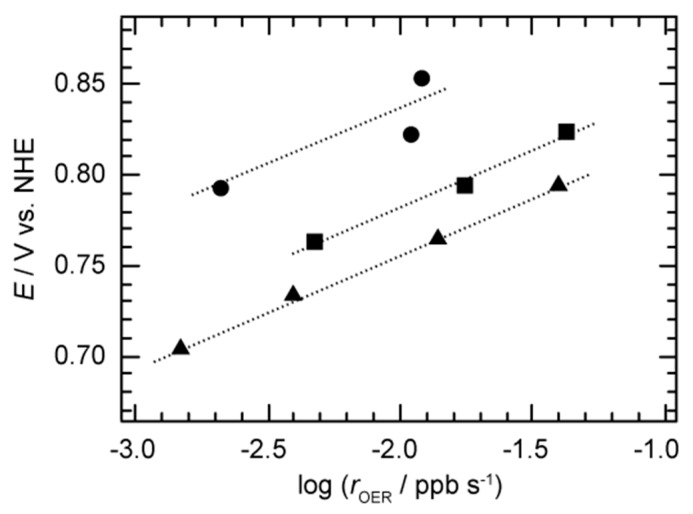
Tafel plot of logarithm reaction rate of OER ($r_{\mathrm{OER}}$) versus electrochemical potential (*E*) under oxygen evolution condition from F–carbon. The data were recorded at pH 13 (circle), pH 13.5 (square), and pH 14 (triangle). Values of $r_{\mathrm{OER}}$ were evaluated from the slope of concentration of oxygen versus time: d$C_{O_{2}}$/d*t*.

To evaluate the pH dependence of $r_{\mathrm{OER}}$, we investigated the $r_{\mathrm{OER}}$ from the measurement of $C_{O_{2}}$ under potentiostatic polarization. In the case of pH 12, $C_{O_{2}}$ was almost same regardless of applied potential (see Figure S5). However, $r_{\mathrm{OER}}$ at pH 13.5 and pH 14 was significantly increased compared to the results of pH 12 and pH 13. In addition, $r_{\mathrm{OER}}$ at the potential of 1.62 V corresponded to a maximum value of 0.038 ppb s^−1^ in KOH solution (pH = 14). Therefore, the OER activity of F–carbon is enhanced by increasing the pH and *E*. In addition, oxygen evolution rate was slightly decreased with a backward trace from 1.62 to 1.53 V in comparison with a forward trace from 1.53 to 1.62 V at pH 13. Figure 3 shows Tafel plots of OER from F–carbon by plotting $r_{\mathrm{OER}}$ and *E* versus NHE scale. The Tafel slope can be treated as same even for $r_{\mathrm{OER}}$ instead of $j_{\mathrm{OER}}$ (vide infra). Tafel slopes were similar regardless of pH of KOH aqueous solutions. Tafel slopes at pH 13, pH 13.5, and pH 14 were 62 mV dec^−1^, 62 mV dec^−1^, and 66 mV dec^−1^, respectively. In addition to that, potential shifts at the same $r_{\mathrm{OER}}$ were quite similar to each other, and ∂*E*/∂log(*a*_H^+^_) was 63 mV pH unit^−1^. This observation suggests that $r_{\mathrm{OER}}$ is related to the activity of protons.

Importantly, observed OER activity was not due to the deposition of metal impurity species [17,18]. In our previous work, we demonstrated that pristine carbon materials show negligible oxygen evolution rates compared to the observed current density [4]. If that comes from the activity of impurity deposition, ignorable activity will not be observed. The Faradaic efficiency was roughly estimated from the conversion of $r_{\mathrm{OER}}$ to corresponding current density by calculation of nearly 100% of Faradaic efficiency for RuO_2_ (Figure S6 and Tables S1–S3). The calculated Faradaic efficiency ranged from 5% to 30% for our F–carbon catalyst. Even though carbon corrodes easily under OER conditions, the materials that did remain showed activity, suggesting that our catalyst has high potential for OER.

### 3.2. Kinetic Model and Theoretical Calculation for F–Carbon

Conventionally, the kinetic model is constructed from rate-limiting kinetics in the net reaction of the catalytic cycle. OER current ($j_{\mathrm{OER}}$) is described by $r_{\mathrm{OER}}$, Faraday’s constant (*F*), and number of electron (*n*). $j_{\mathrm{OER}}$ is expressed as follows:

(1) $$j_{\mathrm{OER}}=nFr_{\mathrm{OER}}$$

Therefore, we can use $r_{\mathrm{OER}}$ as a parameter for Tafel analysis, and Tafel slope is expressed herein as *∂E*/*∂*log($r_{\mathrm{OER}}$). Differences in pH induce a shift of thermodynamic potential between the water and oxygen equilibrium redox couple. Therefore, the order of the proton is estimated from the following Equation (2), which is based on Euler’s chain rule [19,20,21]:

(2) $$\frac{\partial \log(r_{\mathrm{OER}})}{\partial \log(a_{H^{+}})}=-\frac{\partial \log(E)}{\partial \log(a_{H^{+}})}\frac{\partial \log(E)}{\partial \log(r_{\mathrm{OER}})}$$

Here, *∂E*/*∂*log($r_{\mathrm{OER}}$) is a conventional Tafel slope, and *∂E*/*∂*log(*a*_H^+^_) is explained as a potential shift depending on pH at the same $r_{\mathrm{OER}}$. As shown in Figure 3, Tafel slopes for all the plots correspond to almost 59 mV dec^−1^ value, regardless of the pH that corresponds to 2.3 × RT/F. In addition to that, the potential shift of pH at the same reaction rate (*∂E*/*∂pH*) is 60 mV pH unit^−1^. By using Equation (2), $r_{\mathrm{OER}}$ is revealed to be proportional to the inverse first order dependence of proton activity.

The reaction order for $r_{\mathrm{OER}}$ is described by the following expression, using the constant *v*_0_:

(3) $$r_{\mathrm{OER}}=r_{0}{\left(a_{H^{+}}\right)}^{-1}\exp\left(\frac{FE}{RT}\right)$$

The rate expression carries the observed inverse first order dependence on proton activity, and the exponential relationship with electrochemical potential (*E*). Rearrangement of the log form of Equation (3) yields a Tafel slope (*∂E*/*∂*log($r_{\mathrm{OER}}$)) of 59 mV decade, which is also consistent with experimental data. Equation (3) represents a mechanistic sequence involving a reversible one electron, one proton equilibrium step, followed by a rate-determining chemical step. These mechanisms are expressed by Equations (4) and (5) as follows:

(4) $$\frac{\theta }{1-\theta }=K{\left(a_{H^{+}}\right)}^{-1}\exp\left(\frac{FE}{RT}\right)$$

(5) $$r_{\mathrm{OER}}=k_{2}\theta$$

**Figure 4:**
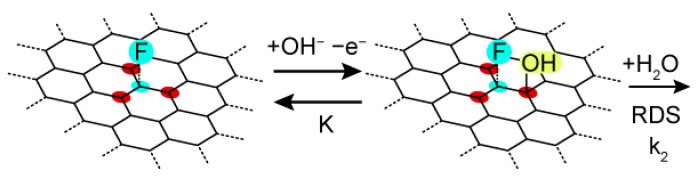
Schematic illustration of the reaction mechanism of F–carbon. Pre-equilibrium of the hydroxide adsorption and one-electron oxidation occurred at positively charged carbon sites (red) with respect to the fluorine-doped carbon sites (blue). The adsorbed hydroxide reacts with H_2_O as the irreversible rate-determining chemical step.

*θ* is the coverage of the adsorption step involving the proton-coupled electron transfer process. Due to the pre-equilibrium, *θ* is expected to be small value. Therefore, *θ*/(1−*θ*) ≃ *θ*. Importantly, the intrinsic exchange reaction rate is determined by pre-factor k_2_K. This suggests that one of following is important factor (1) the strong interaction between surface-adsorbed hydroxide and water or (2) the stronger strong interaction between hydroxide and surface. In order to investigate the structural origin of F–carbon, we conducted a theoretical calculation for the model molecular structure of F–carbon. In our previous study, we found 90.8% of carbon element and 3.9% of fluorine from semi-ionic C–F bonding [4]. The observed value corresponds to one C–F bond site per 50 Å^2^. We constructed an aromatic system based on the model structure (C_42_H_16_F) for structure evaluation (see Supplementary Materials for detail). Importantly, we found that fluorine-doping on the graphene-like sheet induced a positive charge for the neighboring α-carbon atom, revealed by Mulliken charge analysis (see Figure S7). In the case of semi-ionic C–F bonding structures, α-carbon is positively charged with the values of 0.196 and 0.189. On the other hand, in the case of covalent C–F bonding structures, α-carbon is less positively charged at 0.124 and 0.112. This result suggests that fluorine doping induces charge localization at the α-carbon atom, which can be an active site for turnover rate-determining steps as shown in Figure 4. We expect that fluorine-doping induces either (1) the increase of hydroxide adsorption energy or (2) the increase of electrophilicity of adsorbed hydroxide species. Interestingly, observed phenomena in F–carbon are reminiscent of highly active catalysts such as perovskites and surface platinum oxides [20,22,23].

## 4. Conclusions

In conclusion, we showed from the kinetic data profile that our F–carbon possesses high catalytic activity. In most cases, carbon catalysts suffer from decomposition, and mechanistic study is quite difficult in terms of catalyst design. Our method, based on an O_2_ sensor and RDE, enables the in situ monitoring of O_2_ concentration directly from solution. Tafel data and pH dependence proved that OER catalysis proceeds with proton-coupled electron transfer process and an irreversible chemical step. The present study shed light on potential catalysts for designer interfaces.

## Supplementary Materials

The following are available online at http://www.mdpi.com/1996-1944/12/2/211/s1, Figure S1: XPS results of F–carbon around (a) F 1s range and (b) wide scan; Figure S2: Electrochemical cell setup. Three-electrode cell was used for the controlling the electrochemical potential of working electrode. Evolved O_2_ was detected by O_2_ sensor immersed in solution; Figure S3: OER from RuO_2_ in 0.1 M KOH solution (pH = 13). (a) Applied potential (E), (b) current density (j), and (b) O_2_ concentration ($C_{O_{2}}$) were plotted versus time; Figure S4: Chronoamperometry at different electrochemical potentials. Applied electrochemical potentials were shown in above; Figure S5: Time–course analysis of O_2_ concentration trace under the potentiostatic polarization. Applied electrochemical potentials were shown in above; Figure S6: Correlation between rate of OER and current density in RuO_2_ under oxygen evolution condition. Data was adapted from Figure S3; Table S1: Calculation of Faradaic efficiency for F–carbon under OER conditions in pH 13; Table S2: Calculation of Faradaic efficiency for F–carbon under OER condition in pH 13.5; Table S3: Calculation of Faradaic efficiency for F–carbon under OER condition in pH 14; Figure S7: Mulliken charge analysis for semi-ionic C–F model structure and covalent C–F model structure. Green color and red color indicates the positive and negative charges respectively; Appendix: Cartesian coordinates for C_42_H_16_ and C_42_H_16_F (semi-ionic and covalent).
