# Supplementary material for: Revealing High Oxygen Evolution Catalytic Activity of Fluorine-Doped Carbon in Alkaline Media
Source: Materials (Basel). 2019 Jan 10;12(2):211. doi: 10.3390/ma12020211 (PMC6356676; doi:10.3390/ma12020211)
Supplement: Supplementary file 1 [file materials-12-00211-s001.pdf]

Supplementary Materials: Revealing High Oxygen Evolution Catalytic Activity of Fluorine-Doped Carbon in Alkaline Media

Jeheon Kim [1], Tomohiro Fukushima [1], Ruifeng Zhou [2] and Kei Murakoshi [1,*]

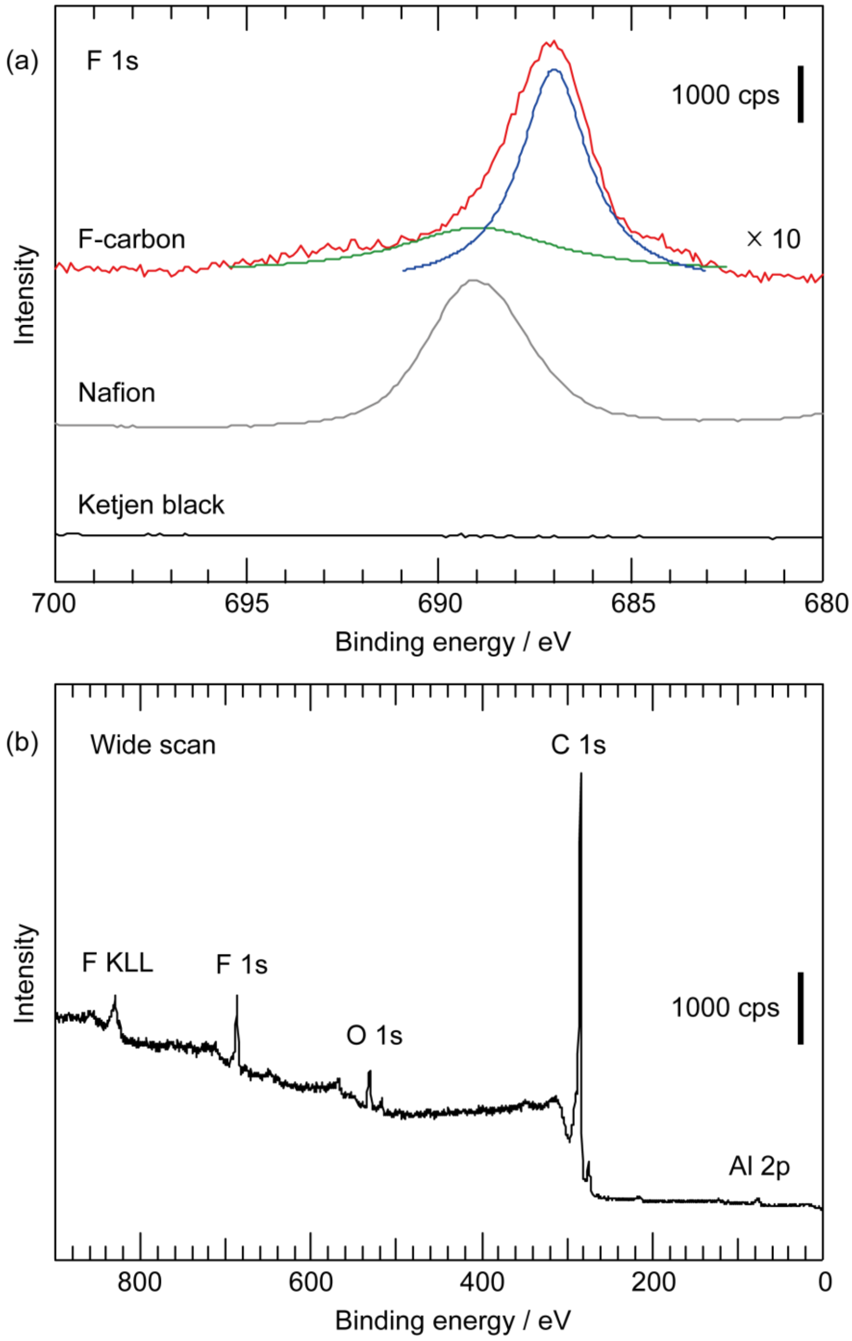

Figure S1. XPS results of F−carbon around (**a**) F 1s range and (**b**) wide scan.

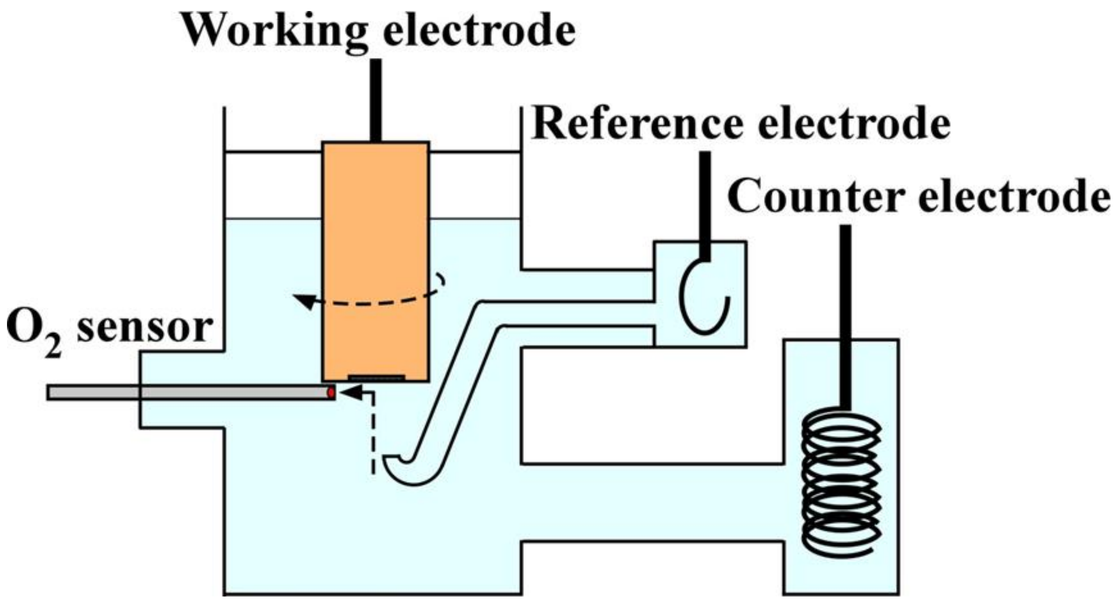

Figure S2. Electrochemical cell setup. Three-electrode cell was used for the controlling the electrochemical potential of working electrode. Evolved O_2 was detected by O_2 sensor immersed in solution.

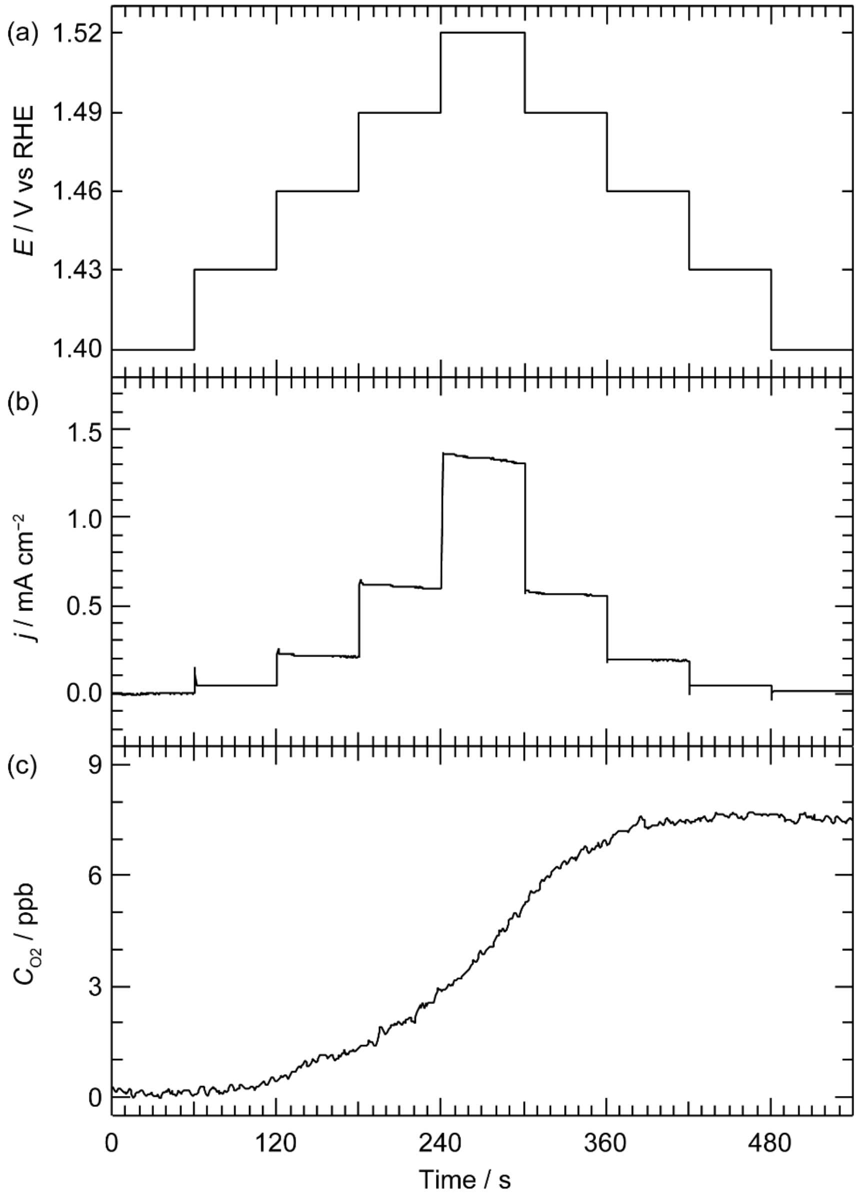

Figure S3. OER from RuO_2 in 0.1 M KOH solution (pH 13). (**a**) Applied potential (*E*), (**b**) current density (*j*), and (**c**) O_2 concentration (C_{O2}) were plotted versus time.

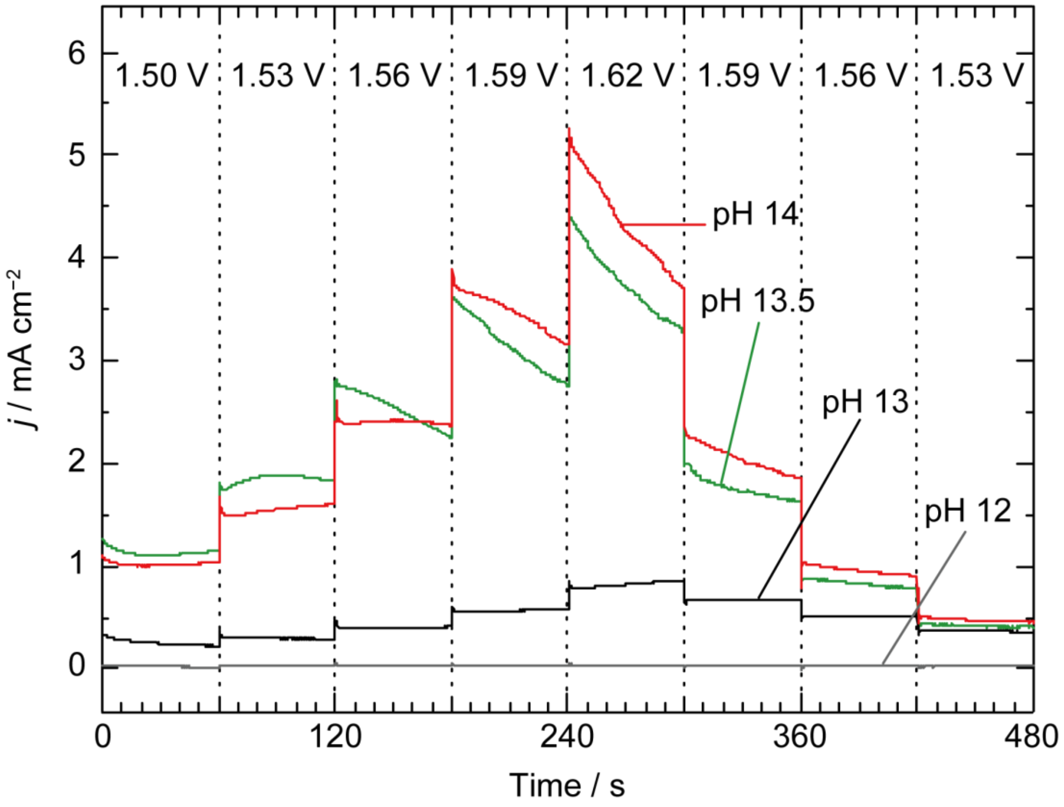

Figure S4. Chronoamperometry at different electrochemical potentials. Applied electrochemical potentials were shown in above.

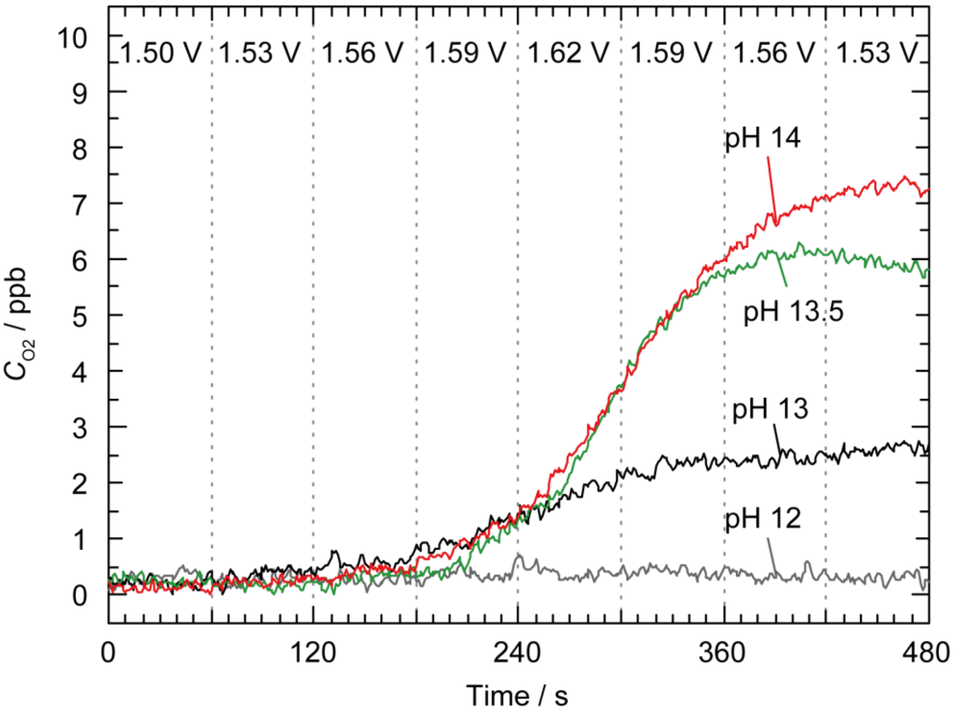

Figure S5. Time-course analysis of O_2 concentration trace under the potentiostatic polarization. Applied electrochemical potentials were shown in above.

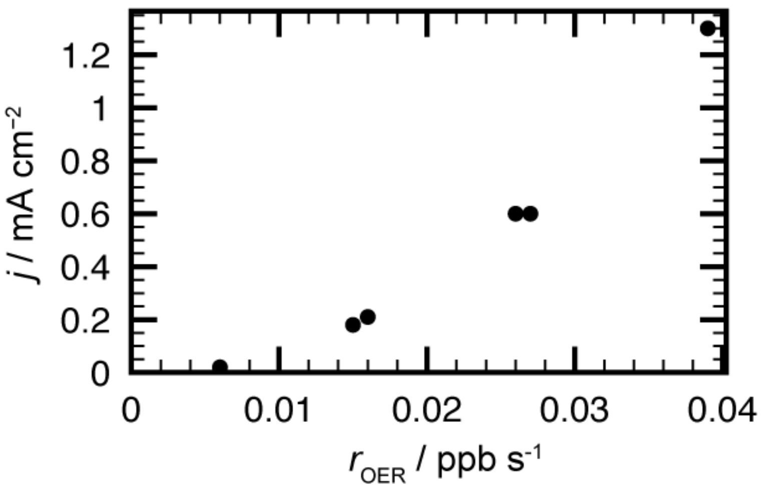

Figure S6. Correlation between velocity of OER and current density in RuO_2 under oxygen evolution condition. Data was adapted from Figure S3.

Table S1. Calculation of Faradaic efficiency for F−carbon under OER condition in pH 13.

E vs. RHE	v_{OER} / ppb s^{-1}	j_{OER} / mA cm^{-2}	j_{total} / mA cm^{-2}	Faradaic Efficiency
1.59	0.011	0.075	0.48	0.15
1.62	0.012	0.12	0.71	0.17

Table S2. Calculation of Faradaic efficiency for F−carbon under OER condition in pH 13.5.

E vs. RHE	v_{OER} / ppb s^{-1}	j_{OER} / mA cm^{-2}	j_{total} / mA cm^{-2}	Faradaic Efficiency
1.59	0.017	0.32	3.1	0.10
1.62	0.042	1.3	3.9	0.33

Table S3. Calculation of Faradaic efficiency for F−carbon under OER condition in pH 14.

E vs. RHE	v_{OER} / ppb s^{-1}	j_{OER} / mA cm^{-2}	j_{total} / mA cm^{-2}	Faradaic Efficiency
1.59	0.014	0.19	3.2	0.058
1.62	0.039	1.2	4.1	0.29

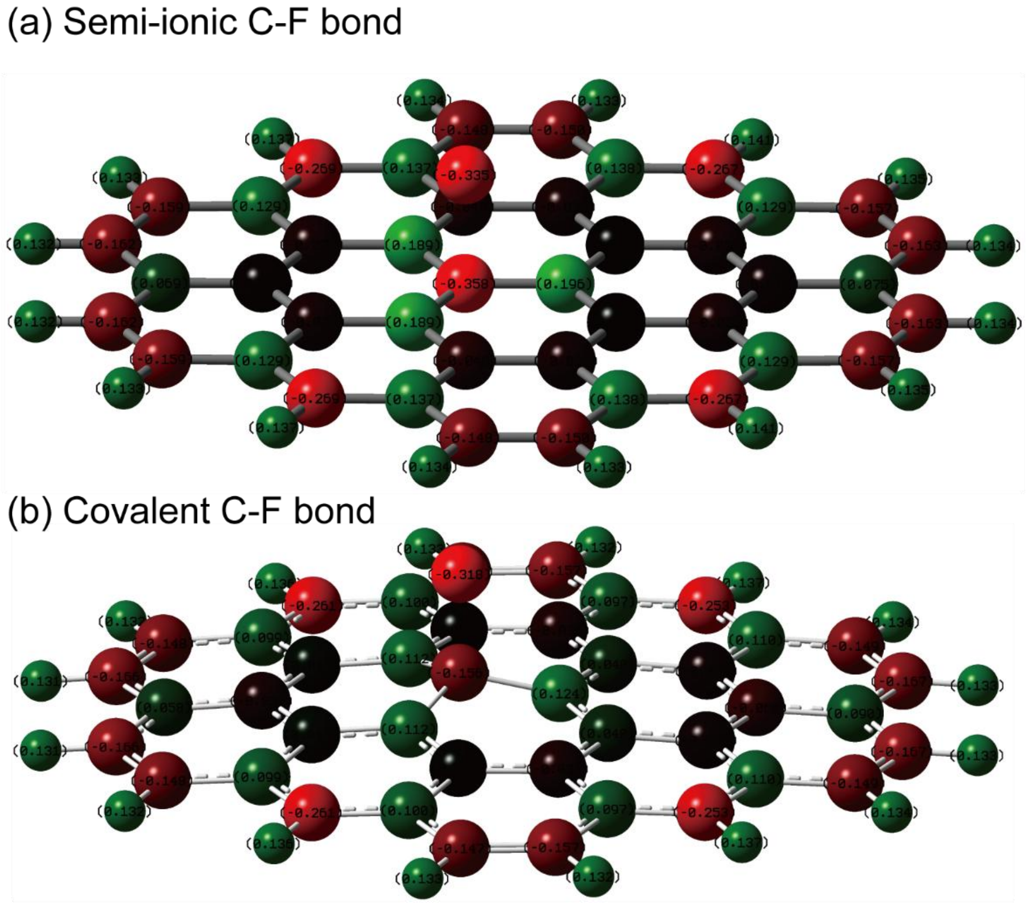

Figure S7. Mulliken charge analysis for semi−ionic C–F model structure and covalent C–F model structure. Green color and red color indicates the positive and negative charges respectively.

Appendix. Cartesian coordinates for $C_{42}H_{16}$ and $C_{42}H_{16}F$ (semi-ionic and covalent).

$C_{42}H_{16}$

Element-Label	x	y	z
C	−4.72378	2.00363	0.07245
C	−3.92364	0.85426	0.09064
C	−2.51742	0.99042	0.08927
C	−1.93125	2.27678	0.06969
C	−4.14435	3.27402	0.05316
H	−5.80843	1.92086	0.07315
C	−2.75127	3.42584	0.05155
H	−4.79432	4.1463	0.03935
C	−4.48835	−0.42728	0.11015
C	−1.6953	−0.15957	0.10748
C	−3.67592	−1.56289	0.12817
H	−5.56873	−0.55415	0.11152
C	−2.27959	−1.44461	0.12708
H	−4.14643	−2.54366	0.14309
C	−0.52484	2.41168	0.06828
C	0.29616	1.26286	0.08639
C	1.70206	1.39899	0.08486
C	2.28736	2.68399	0.06521
C	0.05961	3.69737	0.04868
C	1.46706	3.83365	0.04715
C	−0.28913	−0.02214	0.10602
C	2.52306	0.25016	0.10296
C	0.53117	−1.1718	0.1242
C	1.93862	−1.03552	0.12267
C	3.69353	2.82142	0.06357
C	4.51565	1.67142	0.08162
C	5.92186	1.80758	0.07993
C	6.48658	3.08911	0.06025
C	4.27781	4.10646	0.04389
C	5.67414	4.22473	0.04247
H	7.56696	3.21598	0.05858
H	6.14465	5.2055	0.02743
C	3.92948	0.38507	0.10136
C	6.722	0.65821	0.09796
C	4.74949	−0.76399	0.11944
C	6.14258	−0.61218	0.11749
H	7.80665	0.74097	0.09698
H	6.79255	−1.48446	0.1312
C	−1.45178	−2.57675	0.14506
C	−0.05425	−2.45632	0.14386
H	−1.90246	−3.56758	0.1602
C	2.75965	−2.18385	0.1408
C	4.15432	−2.03392	0.13896
H	4.78676	−2.91987	0.15293
C	−2.15609	4.69577	0.03228
C	−0.76142	4.8457	0.03062

H	−2.78853	5.58172	0.01831
C	2.05248	5.11817	0.02755
C	3.45001	5.2386	0.02617
H	3.90069	6.22943	0.01102
C	0.77389	−3.58609	0.16178
C	2.16369	−3.45151	0.16027
H	2.77741	−4.34967	0.17447
H	0.34392	−4.5853	0.17711
C	−0.16546	6.11337	0.01135
C	1.22434	6.24794	0.00984
H	−0.77918	7.01153	−0.00274
H	1.65431	7.24716	−0.00539

$C_{42}H_{16}F$ Covalent

Element-Label	x	y	z
C	−4.71488374	1.99864703	−0.19818917
C	−3.91451374	0.85264703	−0.12341917
C	−2.51112374	0.99155703	−0.02133917
C	−1.92701374	2.27432703	−0.01125917
C	−4.13570374	3.26814703	−0.20791917
H	−5.79679374	1.91352703	−0.27188917
C	−2.74506374	3.41958703	−0.13042917
H	−4.78276374	4.13903703	−0.28774917
C	−4.47543374	−0.42846297	−0.16924917
C	−1.68356374	−0.15060297	0.01375083
C	−3.65919374	−1.55975297	−0.14887917
H	−5.55294374	−0.55827297	−0.24173917
C	−2.26567374	−1.43542297	−0.07150917
H	−4.12404374	−2.54190297	−0.20316917
C	−0.52182374	2.40509703	0.09721083
C	0.30076626	1.27967703	0.40819083
C	1.69813626	1.39643703	0.13316083
C	2.28603626	2.67785703	0.02028083
C	0.06027626	3.68926703	−0.01449917
C	1.46684626	3.82695703	−0.02091917
C	−0.27769374	−0.00183297	0.12210083
C	2.52465626	0.25034703	0.06436083
C	0.53689626	−1.15961297	0.05475083
C	1.94236626	−1.03210297	0.05928083
C	3.69092626	2.81546703	−0.06452917
C	4.51429626	1.66769703	−0.07054917
C	5.91911626	1.80335703	−0.14545917
C	6.48224626	3.08371703	−0.20753917
C	4.27427626	4.09902703	−0.14287917
C	5.66889626	4.21817703	−0.20840917
H	7.56115626	3.21036703	−0.26387917
H	6.13708626	5.19851703	−0.26598917
C	3.92954626	0.38328703	−0.01953917
C	6.71965626	0.65464703	−0.16246917
C	4.74977626	−0.76550297	−0.05420917
C	6.14134626	−0.61513297	−0.11946917
H	7.80288626	0.73725703	−0.21791917
H	6.79039626	−1.48786297	−0.14358917
C	−1.44040374	−2.56599297	−0.07691917
C	−0.04669374	−2.44389297	−0.02906917
H	−1.88924374	−3.55591297	−0.13794917
C	2.76203626	−2.18140297	0.00164083
C	4.15566626	−2.03464297	−0.03372917
H	4.78643626	−2.92105297	−0.07105917
C	−2.15065374	4.68700703	−0.17334917
C	−0.75815374	4.83478703	−0.13364917
H	−2.78044374	5.57048703	−0.26224917

C	2.05139626	5.10896703	−0.12220917
C	3.44722626	5.23019703	−0.16301917
H	3.89634626	6.21934703	−0.23382917
C	0.77705626	−3.57623297	−0.06288917
C	2.16589626	−3.44697297	−0.04142917
H	2.77755626	−4.34569297	−0.08054917
H	0.34442626	−4.57310297	−0.11528917
C	−0.16368374	6.10031703	−0.21232917
C	1.22489626	6.23633703	−0.20051917
H	−0.77724374	6.99509703	−0.29323917
H	1.65484626	7.23295703	−0.27429917
F	0.25850374	1.24537297	2.10731917

$C_{42}H_{16}F$ Semi-ionic

Element-Label	x	y	z
C	5.67474946	1.251513	−0.174988
C	4.98730446	0	−0.174991
C	3.56288146	0	−0.174997
C	2.84751446	1.238284	−0.174999
C	4.99107846	2.443746	−0.174992
H	6.76097246	1.24605	−0.174984
C	3.56082446	2.480029	−0.174998
H	5.53335746	3.384756	−0.174991
C	5.67475046	−1.251513	−0.174988
C	2.84751446	−1.238284	−0.174999
C	4.99107846	−2.443746	−0.174992
H	6.76097246	−1.24605	−0.174983
C	3.56082346	−2.480029	−0.174998
H	5.53335746	−3.384756	−0.17499
C	1.42362846	1.23926	−0.175004
C	0.70733546	0	−0.175004
C	−0.70740254	0	−0.175004
C	−1.42369554	1.23926	−0.175003
C	0.71199846	2.470825	−0.175005
C	−0.71206554	2.470825	−0.175005
C	1.42362846	−1.23926	−0.175004
C	−1.42369554	−1.23926	−0.175003
C	0.71199846	−2.470825	−0.175005
C	−0.71206554	−2.470825	−0.175006
C	−2.84758154	1.238284	−0.174999
C	−3.56294854	0	−0.174997
C	−4.98737154	0	−0.174991
C	−5.67481754	1.251513	−0.174988
C	−3.56089054	2.480029	−0.174998
C	−4.99114554	2.443746	−0.174992
H	−6.76103954	1.24605	−0.174983
H	−5.53342454	3.384757	−0.17499
C	−2.84758154	−1.238284	−0.174999
C	−5.67481754	−1.251513	−0.174989
C	−3.56089154	−2.480029	−0.174998
C	−4.99114554	−2.443746	−0.174992
H	−6.76103954	−1.24605	−0.174984
H	−5.53342454	−3.384756	−0.17499
C	2.83292946	−3.691571	−0.175001
C	1.43787746	−3.713849	−0.175006
H	3.37888046	−4.631473	−0.175001
C	−1.43794454	−3.713849	−0.175006
C	−2.83299654	−3.691571	−0.175002
H	−3.37894754	−4.631473	−0.175002
C	2.83292946	3.691571	−0.175002
C	1.43787746	3.713849	−0.175006
H	3.37888046	4.631473	−0.175002

C	−1.43794454	3.713849	−0.175006
C	−2.83299654	3.691571	−0.175002
H	−3.37894754	4.631473	−0.175002
C	0.68163746	−4.944104	−0.175011
C	−0.68170454	−4.944104	−0.175012
H	−1.23007854	−5.881687	−0.175016
H	1.23001146	−5.881687	−0.175016
C	0.68163746	4.944104	−0.175011
C	−0.68170454	4.944104	−0.175011
H	1.23001146	5.881687	−0.175016
H	−1.23007854	5.881687	−0.175016
F	−0.70707671	0	1.52499597
